# Caring for Children Amidst Chaos: Guidelines to Maintain Health

**DOI:** 10.1289/ehp.114-a584

**Published:** 2006-10

**Authors:** Adrian Burton

It is a simple fact that young children are those most likely to die during humanitarian crises caused by famine, war, and natural disasters. Relief agencies are keenly aware of this and do their utmost to save as many young lives as possible as well as maintain the standard of health care children enjoyed before disaster hit. A 2006 report by the National Research Council, *Child Health in Complex Emergencies*, suggests, however, that these agencies’ efficiency in terms of safeguarding child health might be increased if a set of common, comprehensive, evidence-based clinical guidelines were available for use by all.

The report defines a complex emergency as a situation of armed conflict, population displacement, food insecurity (which could be caused by extended drought, some other natural disaster, or other circumstances), or some combination of these situations with an associated increase in mortality and malnutrition. In addition, during the acute phase, the mortality rate will be at least double that of baseline.

Currently, the report shows, some agencies have their own guidelines for addressing certain areas of child health during emergencies, but lack them for others. Many, however, use guidelines produced by authorities such as the WHO and UNICEF—guidelines that were produced for stable, noncrisis situations, and that might therefore be less applicable in emergency settings. Still others have a distinct lack of clinical guidelines. In addition, many of those guidelines that do exist have either never been assessed for effectiveness or are aimed at physicians, when it is actually personnel with less medical training—including field-instructed volunteers not formally trained in the care of children—who often take on the bulk of child health care provision. Further, these guidelines may not be in a language local health workers understand.

The report throws down a daunting challenge: to produce a single set of locally adaptable clinical guidelines covering all child health problems likely to be encountered in emergency situations, then tailor them to the different expertise levels necessary and translate them into several different languages. This gargantuan task begs the question of whether this is feasible from the viewpoint of human and financial resources. Do relief agencies have the time, money, and personnel to devote to such a project? What would be the forum for such a venture? Who would provide leadership? Would the fear of surrendering independence hinder the adoption of common clinical guidelines by some agencies? In short—can this be done?

## Why Child Health?

Children under the age of 5 regularly bear the brunt of the death toll associated with complex emergencies. The medical literature eloquently shows the need for special attention to child health in such situations. According to a 4 August 1993 *JAMA* report, of all those who died in the 1991 Kurdish refugee crisis, two-thirds were under 5 years of age. In the 10 April 1993 issue of *The Lancet*, another team reported that during the 1992 Somali famine, 74% of all children under age 5 in displaced persons camps died. Four years later, in the 5 April 1997 issue of *The Lancet*, yet another team reported that in 1996, 54% of all the deaths among refugees from Rwanda and Burundi who fled to eastern Zaire were under the age of 5.

“In today’s conflicts, civilians are the main victims of war, and children bear a disproportionate degree of that burden,” explains Richard Brennan, director of the Health Unit of the International Rescue Committee (IRC), a New York–based relief agency. “This problem is demonstrated no more clearly than in the Democratic Republic of Congo, where a recent countrywide survey showed that almost half of the deaths have been among children under the age of five years. The vast majority fall victim not to violence, but to the silent, indirect health consequences of conflict—diarrhea, malaria, pneumonia, and malnutrition.”

Humanitarian crises are not new or in short supply, and relief agencies have amassed huge experience in dealing with both the acute and postacute phases. *Child Health in Complex Emergencies* shows, however, that despite this experience, no relief agency has developed a complete set of clinical guidelines that would help them deal more efficiently with all the child health problems it might encounter in an emergency situation. Relief organizations were found to be least likely to have formal guidelines in areas such as the management of asphyxia, prematurity, neonatal infection, the diagnosis and management of children with HIV, the treatment of tuberculosis, pediatric trauma, and the diagnosis and management of mental health problems. Some organizations, such as Médecins sans Frontières (MSF), have developed clinical guidelines for dealing with many emergency problems, but the report indicates even they lack strategies for diagnosing and managing persistent diarrhea, neonatal health (for example, prevention of tetanus and asphyxia), and trauma management.

Children under 18 represented 39% of the overall population of the eight countries hardest hit by the December 2004 tsunami

–UNICEF

Most other relief organizations do not have clinical guidelines designed specifically for complex emergency situations. During its work in the field, the IRC uses WHO guidelines or country-specific health ministry guidelines, written for use in stable environments. Save the Children uses much the same. Even the American Academy of Pediatrics and the WHO/UNICEF Integrated Management of Child Illnesses project (which attempts to draw together experience on how to deal with child illness to improve national health policies), which might be considered natural sources of possible guidelines, lack them in many areas.

Further, the report indicates more research is needed on evaluating the efficacy of those guidelines that *are* available. It is not enough to simply have guidelines; they need to be evaluated in the field to see whether they respond to real needs.

“There is a need for a single, comprehensive, and evidence-based set of guidelines targeted to the levels of health care worker commonly providing care to children in emergencies,” says lead report author William J. Moss, an assistant professor in the Department of Epidemiology at the Johns Hopkins Bloomberg School of Public Health. “The lack of child health guidelines is likely to affect the efficiency of the overall care of children in emergencies.”

## One Size Fits All?

It is well established that the major killers of children in emergency situations are usually the same as those that take their toll in stable settings—malnutrition, measles, malaria, and diarrheal diseases, to name a few—hence the use by many relief agencies of WHO or health ministry guidelines developed for stable situations. However, in complex emergencies the numbers of children affected can be much greater as routine health care falters, and new problems can arise, such as mental health impairment caused by stress [see, for example, “Crisis Not Over for Hurricane Victims,” *EHP* 114:A462 (2006)]. Relief organizations could therefore benefit from having clinical guidelines that would help them deliver the best care to individual child patients under difficult circumstances, which in turn would help them protect the wider child community from the increased danger of epidemics.

However, emergencies can vary widely in nature. So, is it even possible that one set of clinical guidelines could be applicable to all situations?

“Several factors relating to the great variability in health care needs confound efforts to develop standards and guidelines for the care of children in complex emergencies,” says Moss. “Nevertheless, care could be improved by a single resource that addresses common health problems.”

Some relief agencies, however, express concern. “It would be very difficult for one size of guideline to fit all,” says Myriam Henkens, international medical coordinator for MSF. “NGOs are organized in different ways, have different personnel and different goals. A comprehensive set of guidelines might be feasible in the very early stages of a crisis as identified by the report, when different organizations are facing the same problems, namely [reducing] mortality. However, once this stage has passed, guidelines would be more difficult to follow as each NGO would start to focus on its specific area of expertise.”

46% of the total population of the Democratic Republic of Congo uses improved drinking water sources . . .

Emma Roberts, humanitarian affairs advisor for Save the Children UK, also expresses concern. “Most NGOs work with ministries of health and where possible will use the guidelines and treatment protocols of the country in which they are working,” she says. “There are various reasons why agencies use different protocols—for some diseases such as malaria, there are issues of resistance to certain treatments—and to get round this you would need guidelines that are country-specific and need to be updated very regularly. Agencies will source drugs from different countries, and these sources may also dictate treatment protocols.” In addition, she says, “Cost of treatment would mean that a one-size-fits-all [policy] would have to be the lowest common denominator to ensure that all agencies and governments could afford to implement the guidelines—is this ethical if there are better, but perhaps more expensive, treatments available for some countries? Best practice recommendations need to be drawn together without making guidelines so rigid that national contexts and protocols become secondary.”

Brennan says that producing a set of guidelines that could be adapted to the specific context of each emergency would be ideal—and possible—but “clearly there would have to be country-specific adaptations to address differences in issues such as local epidemiology, antibiotic resistance patterns, and health worker skills.” In addition, he expresses concern that the report did not address two big challenges of health service provision in humanitarian settings: data collection in noncamp settings (the vast majority of the report’s data on mortality, morbidity, nutritional status, and markers of health system effectiveness was from camps, where such data are usually relatively easy to collect) and the need to address problems with health systems in order to scale up services and increase access to health care. “I don’t think that the recommendations address these issues adequately,” Brennan says. “As the report rightly notes, rates of morbidity and mortality in postemergency camps are usually quite low, because people usually have access to health and other essential services. But in protracted emergencies and postemergency situations in noncamp settings, health systems are usually poorly functioning, and people frequently have poor access to health care—especially after the acute emergency has faded and funding levels drop. We have to develop better systems to scale up access to essential services in these settings.”

## Coming Together

The very act of coming together to draw up these guidelines would be a task requiring a large input of resources from scores of relief agencies. “Getting agreement would be expensive on time and resources as it would require multiple layers of consultation with organizations all over the world,” says Roberts. “There is also the question of who should be consulted—and then who within governments should also be consulted.”

. . . and 7% of children under 5 with diarrhea receive oral rehydration and continued feeding

–UNICEF

She suggests a further hurdle would simply be to get enough NGOs to engage in the project, a requirement if it is to be a truly inclusive process rather than a U.S.- or Eurocentric exercise, as previous initiatives, she contends, have often been. Brennan believes another major hurdle would be to get the different agencies to commit their time.

However, if experts did convene, they would not be starting from scratch since different agencies might be willing to make the guidelines they currently use available for discussion. “Much of this work has already been done and there is no need to reinvent the wheel,” says Brennan. “Plenty of good reference documents already exist.”

MSF, for example, already makes some of its guidelines available on the Internet. However, one of the major technical difficulties would be deciding which elements to pick and choose from the guidelines available since none of the relief agencies questioned had any formal process for evaluating those they use. Decisions would therefore need to be taken on the basis of expert opinion, and assessment systems put in place to see whether those chosen were actually up to the job. This would require further resources and possible revision of certain guidelines.

Finally, there is no guarantee that any guidelines recommended would be adopted by all relief agencies. “MSF would also be concerned about the potential loss of our independence,” explains Henkens. “In emergency situations the argument for greater coordination between NGOs is a strong one, but this cannot come at the expense of responding quickly and effectively on the basis of need alone.”

## Precedents

Although the problems facing a project to produce comprehensive clinical guidelines would be difficult to overcome, precedents exist in other arenas of standard setting that suggest such a venture could be successful.

The Sphere Project, an initiative of NGOs and the International Red Cross and Red Crescent movement, was launched in 1997 to develop a humanitarian charter defining the rights of populations affected by disaster, and to identify the minimum standards to be attained in disaster assistance, based on agencies’ experience in four areas: water, sanitation, and hygiene promotion; food security, nutrition, and food aid; shelter, settlement, and nonfood items; and health services. This project, involving at least 400 organizations in 80 countries, led to the production of a handbook in 2000, with a revised edition for 2004. This resource (available for free online in English, French, Arabic, Russian, and Spanish, as well as several locally translated versions) is designed to be used all over the world and in most common disaster settings.

Although the handbook advances no strict ways of reaching these targets, it does offer indicators by which relief agencies can test whether they attain them, and lists questions that need to be addressed when applying the standards and indicators in specific contexts. Alison Joyner, Sphere Project manager, says effective use of the handbook should enable organizations to see whether they have properly maintained the dignity of those who receive their aid.

Many NGOs now use the Sphere Project charter and minimum standards to guide their interventions in emergency situations. “The challenge was, and remains to some extent, to communicate the correct messages clearly, and to establish a consensus on what could be agreed in terms of standards and indicators,” explains Joyner. She adds that persevering with making the case, and answering criticisms as honestly and constructively as possible, was a key means by which the project tackled criticisms from groups that challenged the possibility of delineating “universal” standards. “Even those organizations who were, and some of whom remain, critical of Sphere, were involved to a greater or lesser extent in the initial establishment of consensus,” she says.

34% of Gulf Coast children living in FEMA housing have at least one diagnosed chronic medical condition

–National Center for Disaster Preparedness

Joyner points out that there is very little that is new in Sphere—it brings together in one place what already existed in the many guidelines that are referenced at the end of each chapter. “The handbook should help individuals and organizations to achieve better quality and accountability in their work—this is the aim of the project—particularly from the point of view of those affected [by disaster],” she says. There is still one major drawback to the handbook, however. “Unfortunately,” says Joyner, “we still have little concrete evidence to demonstrate to what extent it does support better quality and accountability in humanitarian response, but collecting and collating such evidence is a priority of the current and coming work plans.”

Along similar lines, the Inter-Agency Network for Education in Emergencies, which was conceived in 2000 during the World Education Forum’s Strategy Session on Education in Emergencies in Dakar, has the remit to improve interagency communication and collaboration within the context of education during emergencies. The organization describes itself as an open network of 300 UN agencies, NGOs, donors, practitioners, researchers, and individuals from affected populations that work together to ensure the right to education in emergencies and postcrisis reconstruction. The network guidebook is a capacity-building and training tool for governments and international agencies, helping them improve their contribution to ensuring education during emergencies. Although not a health-based document, the guidebook does serve as an example of what can be achieved when organizations pool resources and pull together.

## Leadership

Roberts points out that for comprehensive clinical guidelines to be produced, a very strong leadership team whose members are recognized as health experts by all stakeholders would be essential. “WHO would be the most appropriate organization to lead on child health,” she says, adding, “Save the Children would be interested in actively participating and contributing to approaches to improve the effectiveness of child health programs in complex emergencies.”

Brennan agrees. “The IRC would be willing to be a member of a committee [involved in guideline production]—but leadership has to come from WHO and UNICEF.”

Indeed, the WHO is already adopting a lead role in this process, says Ala-Dinabdul Sahid Alwan, representative of the WHO Director-General for Health Action in Crises. “Child health in complex emergencies and in crisis situations is indeed a priority area of work. Maternal, newborn, and child health is now listed as one of the [main priority areas of work] that are automatically addressed in any emergency,” Alwan says. “There is already a WHO-led process under way to develop [a manual of clinical guidelines]. We are expecting the first draft of the manual to be circulated in the coming months.” Once this draft becomes available, it will form the basis for major consultation, field testing, and further review, the normal process for any new WHO technical publication. Alwan adds that the Inter-Agency Standing Committee Health Cluster led by the WHO would provide an excellent forum for UN agencies, the Red Cross, and NGOs to consult on priorities, gaps, and approaches so that they can be handled in a concerted and efficient way.

To move any new guidelines from draft to practice will, however, require goodwill and the input of resources—financial, intellectual, and technical—both from the WHO and from the parties it is hoped will use such guidelines. Organizations that have developed many of their own guidelines—and therefore are ahead of the game—are in an excellent position to bring ideas to the table, but may need convincing that they should adopt anything new. And certainly it will be no easy task to produce guidelines adjusted for the different levels of health care worker involved in emergency child health work, especially since these may differ in different countries.

A ready reference of guidelines could take years to complete, and even then frequent changes to it would be required as the arduous task of assessing its content in the field is undertaken. The struggle is certainly uphill, but the goal of saving children’s lives and relieving their suffering in complex emergencies could be no more noble.

## Figures and Tables

**Figure:**
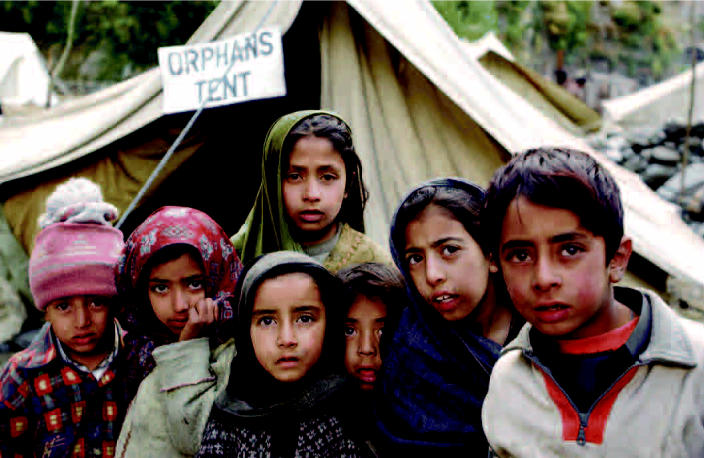


**Figure:**
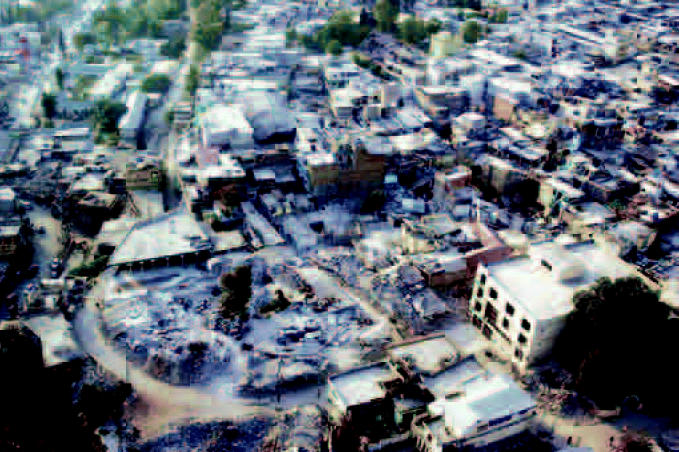
Pakistan Children orphaned by the 8 October 2005 earthquake wait outside a medical tent set up by the Pakistan Army in Chham. (above) Aftermath of the 7.6-magnitude quake in Muzaffarabad that killed 73,000 people and left 3 million homeless. Acute malnutrition in the area, estimated at 5–10% prior to the quake, has been exacerbated by the disaster.

**Figure:**
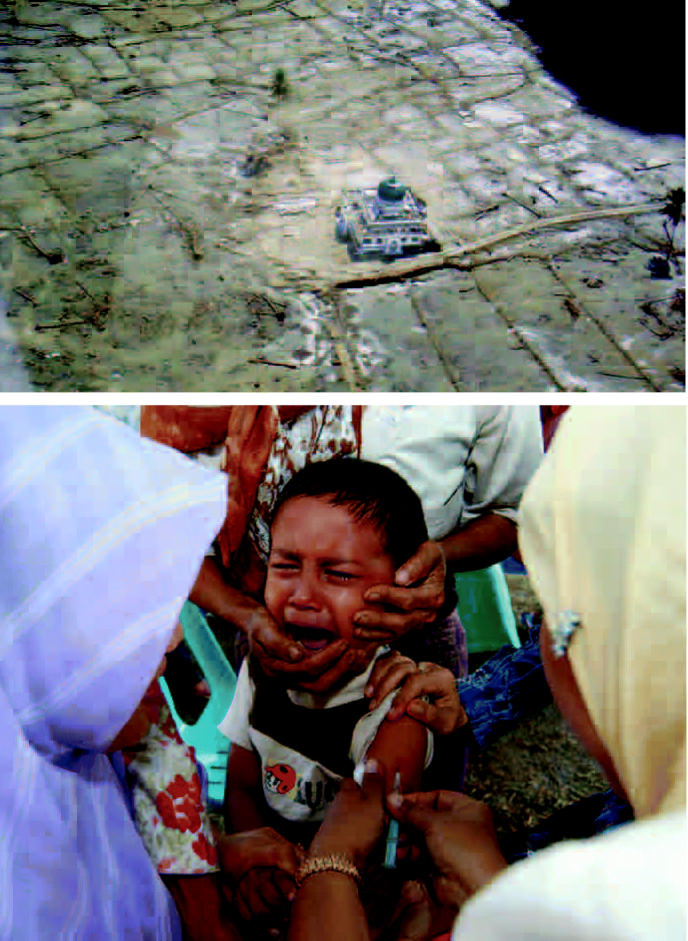
Indonesia (clockwise from above) A mosque is all that’s left standing in Aceh, Indonesia, following the December 2004 tsunami that killed more than 100,000 people and left more than 1 million homeless. A doctor with the International Rescue Committee treats a young boy in Paya Seumantok. A child is vaccinated to prevent infectious disease, a major concern following the tsunami.

**Figure:**
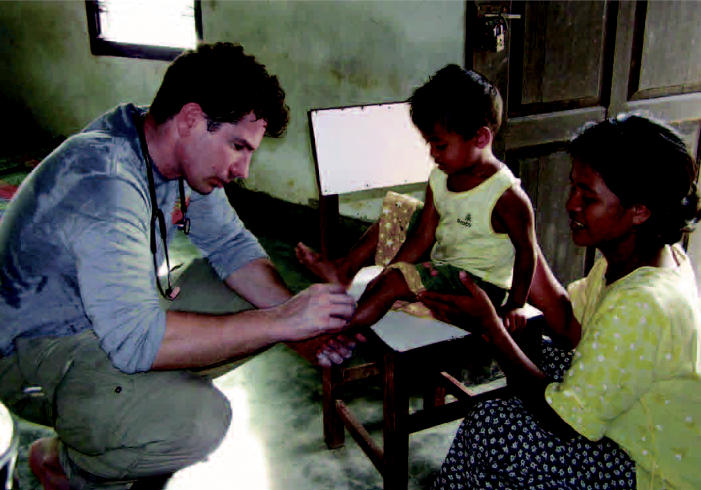


**Figure:**
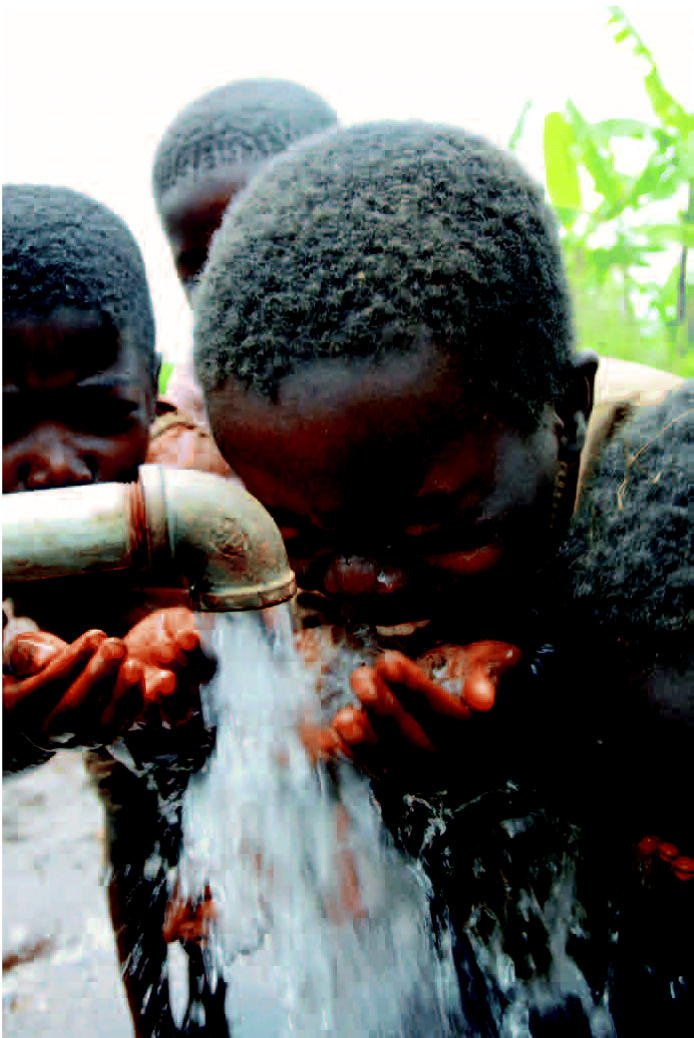


**Figure:**
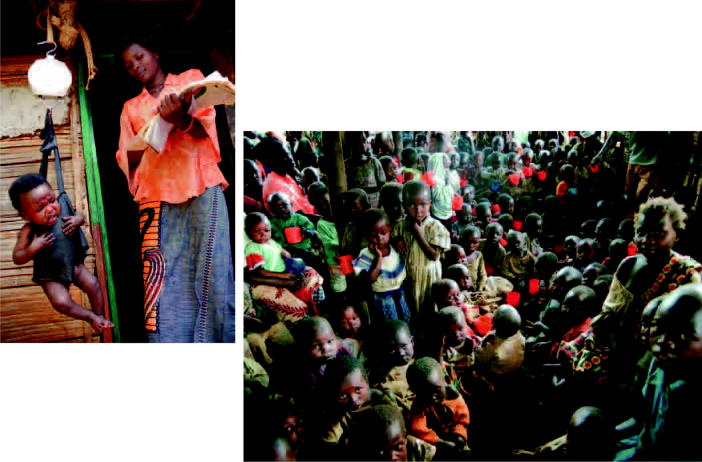
Democratic Republic of Congo Children drink from a communal well built in hopes of bringing together two communities near Bukavu that have had a tense relationship in the past. A nurse weighs a baby in Kabare as part of a program that provides health services to 65,000 children under the age of 5. A feeding center in Kavumu.

**Figure:**
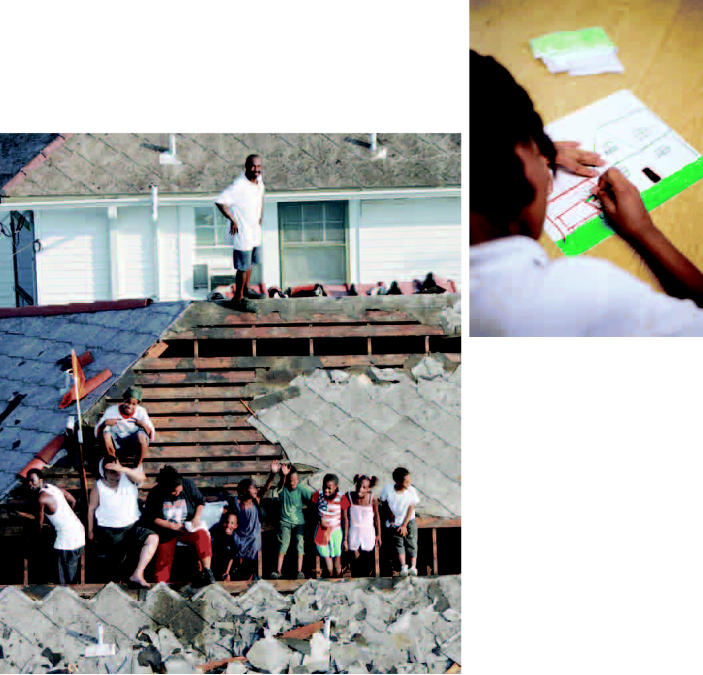
United States (left) New Orleans residents wait to be rescued from the floodwaters of Hurricane Katrina, which hit on 29 August 2005, leaving thousands of residents homeless. (above) A child draws as part of Save the Children’s Psychosocial Structured Activity Program following Katrina; children were asked to draw an image that made them feel safe.

**Figure:**
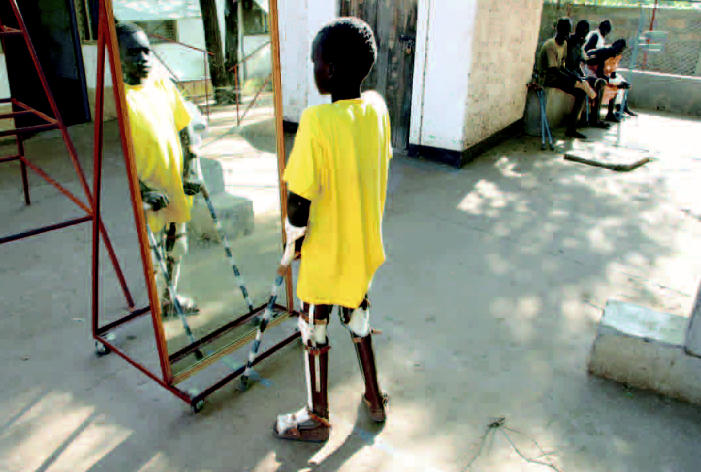
Kenya A boy learning to walk again examines his reflection in a mirror at Lopiding hospital, which was established to care for patients wounded in the ongoing civil war in Sudan.

